# Correlation between mean body mass index in the population and prevalence of obesity in Brazilian capitals: empirical evidence for a population-based approach of obesity

**DOI:** 10.1186/s12889-015-1637-1

**Published:** 2015-04-02

**Authors:** Jackeline Christiane Pinto Lobato, Pauline Lorena Kale, Luis Guillermo Coca Velarde, Moyses Szklo, Antonio José Leal Costa

**Affiliations:** Federal University of Rio de Janeiro – Institute of Public Health, Rio de Janeiro, Brazil; Department of Statistics, Fluminense Federal University, Rio de Janeiro, Brazil; Department of Epidemiology, Johns Hopkins University, Baltimore, USA

**Keywords:** Geoffrey Rose, Population approach, Epidemiology, Obesity, Body mass index

## Abstract

**Background:**

The purpose of this study was to investigate the association between mean body mass index (BMI) and prevalence of obesity in adult populations living in Brazilian State capitals.

**Methods:**

An ecological study was conducted, using data from the National Household Budget Survey conducted in July 2002 through June 2003, including a representative sample of 48.470 households. Pearson’s correlation and linear regression coefficients were estimated in order to define the relationships of mean BMI and sex-specific, age standardized obesity prevalence (BMI ≥ 30.0 kg/m^2^) in adults aged 20 to 59 years.

**Results:**

Stronger correlations between BMI and prevalence of obesity were observed in women (r = 0.9; p < 0.001) than in men (r = 0.6; p = 0.001) in all analyzes. A reduction of one unit in mean BMI predicted a decline in the prevalence of obesity of about 4.0% (95% CI: 1.7 - 6.3) in men, and 3.4% (95% CI: 2.6 – 4.3) in women.

**Conclusion:**

We found a correlation between BMI and prevalence of obesity, particularly among women, suggesting that population-based strategies would be effective to reduce the prevalence of obesity in adult populations living in Brazilian state capitals.

## Background

Obesity has become a public health problem worldwide and, thus, it is imperative that strategies for its prevention and control be implemented. Overweight and obesity prevalence in Brazilian adults in 2012 were 51.0% and 17.4%, respectively [1]. As environment influences nutritional status, intervention at the individual level alone may not be effective in preventing or reducing obesity [2-4].

The different prevention strategies presented by Rose (high-risk and population-based) are still subject to debate [5]. In general, large health benefits are derived from population-wide interventions, although individual gains may be disappointingly trivial [5,6]. Rose & Day’s study results [7] strongly suggest that public health interventions should not only target obese individuals, but also aim at the entire reference population in order to shift the body mass index (BMI) distribution in a favorable direction.

Among the published studies on the population-based approach proposed by Rose [8-11], scarce data are available regarding the relationship of BMI distribution to obesity prevalence in developing countries. Focusing on the social, cultural, political and physical environments influencing nutritional status is a potential way to reduce the prevalence of obesity [12].

In Brazil, the National Food and Nutrition Policy [13] promotes healthy diets and active lifestyles, and defines a set of actions to ensure environments that support these practices, in line with the Global Strategy for Diet, Physical Activity and Health [14]. In Brazil, in the last two decades, there were some advances in promoting health such as the regulation of unhealthy food marketing, the promotion of local agricultural production and of professionally oriented free physical activities in several cities [15]. However, as obesity prevalence has been continuously rising, additional population-based policies are needed in order to control this epidemic [15].

Using a population-based approach, the aim of the present study was to investigate the association between mean BMIs and point prevalence rates of obesity in the adult populations living in Brazilian State capitals.

## Methods

A study was conducted ecologically correlating BMI with obesity in the adult populations from twenty-six Brazilian State capitals.

### Study population

Data were obtained from the National Household Budget Survey (HBS; ‘Pesquisa de Orçamentos Familiares’) conducted by the Brazilian Institute of Geography and Statistics (IBGE) from July 2002 through June 2003. A nationwide probability sample of 48.470 households was selected using a two-stage cluster sampling design, with stratification by rural/ urban areas and average household schooling levels. The primary sampling units were selected by systematic sampling proportional to the number of households in each census tract. Households were selected by simple random sampling. Household interviews were conducted over a twelve-month period. The sample included the State’s adult populations aged 20–59 years, and was designed to provide representative estimates at the national, regional, state and capital levels [16].

### Exposure and outcome

Mean BMI (weight/height^2^) and its standard deviation, and the prevalence of obesity (BMI ≥ 30.0 kg/m^2^) [12] were calculated by sex, for each Brazilian State capital. Height and weight were measured, respectively, to the nearest 0.5 cm using a wall-mounted stadiometer, and a calibrated digital scale with a maximum capacity of 150 kg and 100 g precision. The sex-specific age-standardized prevalence of obesity was calculated for each state capital, using as standard weights the Brazilian adult population in 2003 categorized into ten-year age groups. As BMI values below 13.0 kg/m^2^ and above 50.0 kg/m^2^ are likely due to measurement error, they were excluded. Pregnant and breast-feeding women were also excluded.

### Statistical analysis

Exploratory data analysis procedures were used (scatter plots, distribution curves, and measures of central tendency and dispersion). Given the linear correlation between mean BMI and obesity prevalence in our data, Pearson’s correlation coefficient was estimated. A simple linear regression model provided estimates of the obesity prevalence average variation associated with a one unit reduction in mean BMI [17]. As sex and age may be confounding variables, analyses were performed according to sex and age-group (20–39 years and 40–59 years), representing, respectively, young adults and adults. Associations were also investigated using BMI means after excluding obese individuals (‘non-obese’ mean), in order to eliminate the influence of higher BMI values [6,7]. Statistical significance, set at 0.05 (two tailed), was tested, and 95% confidence intervals were estimated.

All analyses were performed using SPSS version 13.0, taking into account the complex sampling design of the National Household Budget Survey.

This study was approved by the Research Ethics Committee of the Institute of Public Health Studies of the Federal University of Rio de Janeiro - IESC/UFRJ. In Brazil, all censuses and surveys are conducted by the Brazilian Institute of Geography and Statistics under the Federal Law number 5534 from November 14, 1968, which guarantees strict secrecy of personal information gathered, and its use only for statistical purposes.

## Results

Data from 26 Brazilian capitals were analyzed. The capital of Tocantins (Palmas) was excluded due to its unstable population structure, which is characterized by an excess of males. The mean BMI for males was 24.9 ± 4.0 kg/m^2^ ranging from 23.8 kg/m^2^ in Salvador (Northeast) to 25.7 kg/m^2^ in Cuiabá (Midwest). Among women, the mean BMI was 24.1 ± 4.5 kg/m^2^, with minimum and maximum vales of, respectively 22.6 kg/m^2^ in Florianópolis (South) and 25.1 kg/m^2^ in Recife (Northeast) (Table 1). When we removed the obese subjects, ‘non obese’ mean BMI was 24.1 ± 2.9 kg/m^2^ for males and 23.0 ± 3.1 kg/m^2^ for females (not shown in a table).

**Table 1:** **BMI means in adult populations (20 to 59 years) in 26 Brazilian capitals by sex, 2002-2003**

			Mean BMI*(%)	
**Region**	**States**	**Capital**	**Men**	**Women**
	Rondônia	Porto Velho	25.4	24.8
	Acre	Rio Branco	24.6	24.0
North	Amazônia	Manaus	24.9	23.8
	Roraima	Boa Vista	25.2	24.8
	Pará	Belém	24.4	23.7
	Amapá	Macapá	25.3	23.8
	Maranhão	São Luiz	24.2	23.1
	Piauí	Teresina	24.5	24.0
	Ceará	Fortaleza	24.8	24.0
	Rio Grande do Norte	Natal	24.9	24.6
Northeast	Paraíba	João Pessoa	25.3	24.7
	Pernambuco	Recife	25.1	25.1
	Alagoas	Maceió	24.6	24.1
	Sergipe	Aracaju	24.5	23.6
	Bahia	Salvador	23.8	23.8
	Minas Gerais	Belo Horizonte	25.0	24.1
Southeast	Espírito Santo	Vitória	25.0	24.6
	Rio de Janeiro	Rio de Janeiro	25.3	24.3
	São Paulo	São Paulo	24.9	24.4
	Paraná	Curitiba	25.3	24.5
South	Santa Catarina	Florianópolis	24.3	22.6
	Rio Grande do Sul	Porto Alegre	25.5	24.6
	Mato Grosso do Sul	Campo Grande	25.3	24.3
Midwest	Mato Grosso	Cuiabá	25.7	24.8
	Goiás	Goiânia	25.0	23.4
	Distrito Federal	Brasília	25.2	23.6
Total			24.9	24.1

*Body Mass Index.

**Figure 1:**
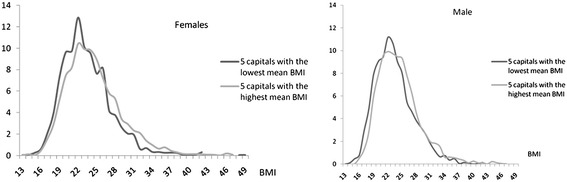
**Body mass index (BMI) distribution of adult populations (20 to 59 years) living in the five capitals with the lowest BMI means and highest BMI means by sex, Brazil, 2002–2003.**

Figure 1 shows the BMI distributions by sex of adult populations living in the five capitals with the lowest and the five with the highest mean BMI values, respectively. All distributions are skewed to the right, although to a slightly lesser degree for males and for the capitals with lowest mean BMI. For the five capitals with higher mean BMIs, a greater flattening of the end tail of BMI distributions is observed than in those with lower mean BMIs.

**Figure 2:**
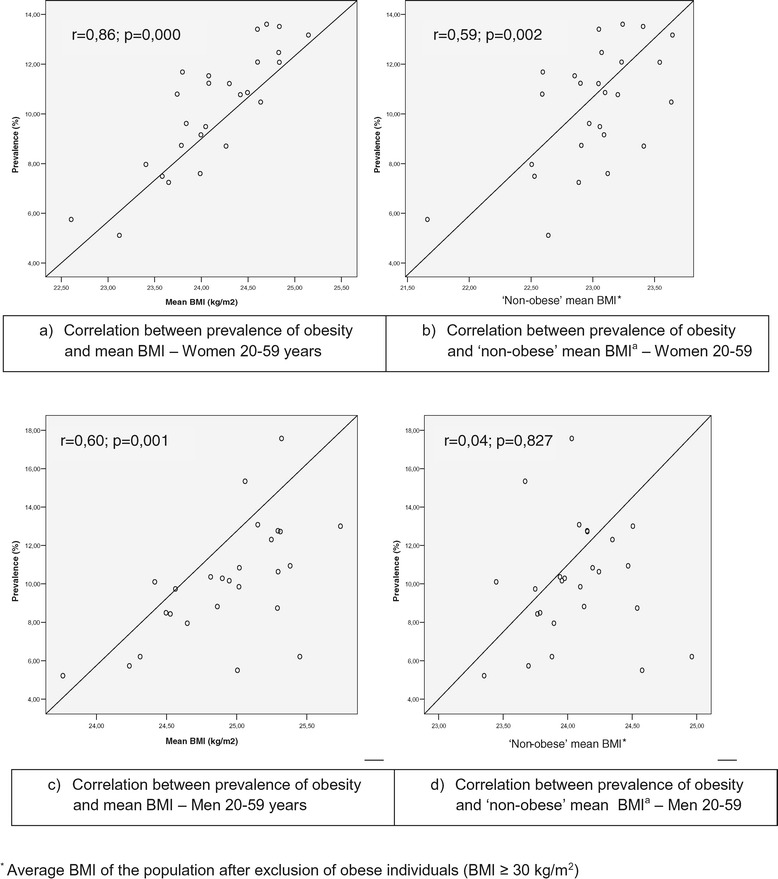
Panels presenting the correlation between mean BMI and prevalence of obesity in adult populations (20 to 59 years) living in 26 Brazilian capitals, by sex, 2002–2003 (panels a and c), and between “non-obese” mean BMI* and prevalence of obesity in adult populations (20 to 59 years) living in 26 Brazilian capitals, by sex, 2002–2003 (b and d). *Average BMI of the population after exclusion of obese individuals (BMI ≥ 30 kg/m2).

The correlation between mean BMI and obesity prevalence for the total population was stronger and highly statistically significant in women (r = 0.9, p < 0.001) than in men, in whom it was also statistically significant (r = 0.6, p < 0.001) (Figure 2 and Table 2).

**Table 2:** **Association of mean BMI (‘non-obese’** ^*^ **and total) and prevalence of obesity in the adult population in the adult population (20–59 years) living in 26 state capitals by sex and age group, Brazil, 2002-2003**

**BMI**	**Age group (Years)**		**Male**				**Female**		
		**r** ^**Ŧ**^	**p-value**	**b**_**1**_ **×100** ^**ǂ**^	**CI 95%** ^**¶**^	**r** ^**Ŧ**^	**p-value**	**b**_**1**_ **×100** ^**ǂ**^	**CI 95%** ^**¶**^
**Total mean**	20-39	0.4	0.040	2.3	0.1 - 4.4	0.9	<0.001	3.4	2.5 - 4.3
**“Non-obese” mean** ^*^		−0.1	0.604	- 0.7	- 3.2 - 1.9	0.2	0.439	0.9	- 1.4 - 3.1
**Total mean**	40-59	0.8	<0.001	5.7	3.7 - 7.8	0.9	<0.001	5.5	4.3 - 6.6
**“Non-obese” mean** ^*^		−0.2	0.445	- 2.0	- 7.1 - 3.2	0.3	0.222	2.5	- 1.6 - 6.7
**Total mean**	20-59	0.6	0.001	4.0	1.7 - 6.3	0.9	<0.001	3.4	2.6 - 4.3
**“Non-obese” mean** ^*^		0.0	0.827	0.4	- 3.1 – 3.9	0.6	0.002	3.3	1.4 - 5.3

*Average Body Mass Index of the population after exclusion of obese individuals (BMI ≥ 30 kg/m^2^).

^**Ŧ**^r. Pearson correlation coefficient.

^**ǂ**^b_1_ coefficient values indicate the average variation in the prevalence of obesity associated with a one unit reduction of the population mean BMI.

^**¶**^95% confidence interval.

After excluding obese individuals from the calculation of the mean BMI, a moderate correlation was seen only in women (r = 0.6, p = 0.002). When correlations were examined by age, the patterns were similar to those seen for the total population when obese individuals were not excluded, although the correlation was found to be stronger for males aged 40–59 years vis-à-vis all males (Table 2).

A one unit decrease in mean BMI was found to be associated with a 4.0% and 3.4% lower obesity prevalence in both men and women, respectively. When obese individuals were excluded, a significant relationship between “non-obese” mean BMI and obesity prevalence was seen only for women, in whom a one unit reduction in mean BMI was related to a 3.3% decrease in obesity prevalence (Table 2). Significant and positive correlations were observed in all age groups and both sexes. In both sexes, ages 40–59 years showed stronger associations (5.7%) than those seen in ages 20–39 years (change in obesity prevalence associated with a one unit decrease in mean BMI = 2.3% for men and 3.4% for women). When obese individuals were excluded, no significant associations or correlations were seen, regardless of sex and age.

## Discussion

According to Rose & Day [7] as the distribution of a particular health related characteristic in a population shifts up or down as a whole, while keeping its dispersion unchanged, the mean and the prevalence of extreme values will be correlated. The observed association indicates how much the mean value of an attribute can predict the prevalence of the extreme values. Thus, the more the BMI distribution is displaced towards higher values for the total population, the higher the prevalence of obesity, and vice-versa. In the present study, the mean BMI of both male and female adult populations of 26 Brazilian capitals was positively correlated with obesity prevalence.

With the exclusion of obese individuals from the calculation of mean BMIs, the correlations were weak or null, except among women, for whom a moderate correlation was observed. Weaker correlations following the exclusion of higher BMI values suggest that the variations in obesity prevalence in the adult populations of Brazilian capitals are likely related not only to the displacement of BMI distributions, but also, as expected, to the skewing of the curves towards high values, a finding that has also been seen in other populations [6]. A previous study suggested that, when the mean BMI in a population increases, there is a disproportionate increase in the amount of obese individuals as well as of those with hypercholesterolemia, in a way that is similar to the relationship between average salt intake and hypertension [6]. The results from our study indicates that, in Brazilian state capitals, obesity in adults should be understood not only as a problem restricted to high risk groups, but also as one that pervades the entire population.

In a study that assessed the effectiveness of two prevention strategies (high-risk and population-wide) to control high blood pressure and serum cholesterol, Emberson et al. [10] observed that a shift of only 5% in the population distribution of both conditions would lead to a 26% reduction in the occurrence of cerebrovascular disease over 10 years. In the same study, the authors found that for the high risk strategy to be more effective, it should focus on a larger portion of the population, resulting in a high number of individuals using at least three drugs to control blood pressure and cholesterol. Laaser et al. [9] applied both prevention strategies to the German population in an intervention study and estimated a 9% reduction in the prevalence of obesity following a one unit decrease of mean BMI in the population.

A longitudinal study of Australian women estimated that a reduction of one unit of BMI of the entire population would reduce the incidence of systemic hypertension in 10% and of diabetes mellitus in 13%. If the intervention encompassed only obese individuals, the expected reductions would be 7% and 17%, respectively. Alternatively, the authors used a strategy called ‘middle road’, that is, an intervention restricted to individuals who were in the right half of BMI distribution in the population (BMI ≥ 24 kg/m^2^), thus avoiding the risks associated with any increase in the number of individuals with low weight. This strategy proved to be the most effective, with potential reductions of 12% and 23% in the hypertension and diabetes incidence, respectively [11]. Evaluation of BMI distributions in populations is important not only to identify epidemiological profiles, but also to help in the choice of the most appropriate interventions and to monitoring effectiveness.

In our study, in adult populations living in Brazilian State capitals, a one unit decrease in the population mean BMI was associated with a decrease in obesity prevalence ranging from 2.3% to 5.7%. This finding strengthens the evidence supporting the understanding of obesity as a response to an “obesogenic” environment, the control of which requires population-wide strategies. To the extent that it promotes high energy intake and reduced physical activity, the environment has an important role in the increasing prevalence of obesity [3]. Living in locations that facilitate and promote physical activity, access to healthy foods, leisure activities and reduced commuting time are some environmental factors that have been shown to be inversely associated with obesity [3]. The understanding of the influence of “built environments” on obesity can be the basis for policy formulation aimed at the population level [3,4]. One of the major weaknesses of the high risk strategy applied to obesity control relates to its limited effectiveness, as it implies the need to identify and treat all obese individuals in the target population. On the other hand, population-wide strategies, such as the reduction of saturated fat and salt contents in processed foods, access to healthy eating habits and promotion of physical activity and leisure opportunities, have the potential of benefiting the population as a whole.

Among the limitations of the present study are the possibility of measurement or classification error and the assumption that adult populations of the 26 Brazilian state capitals would be exposed to similar environmental determinants of obesity. For example, the regression model used did not take into account socio-economic status, such as schooling and per capita income. Thus, future studies should consider not only age and sex, but also other determinants of the outcomes that are also related to the exposure.

Notwithstanding these limitations, our analyses may serve as a feasible model to evaluate the impact of population-based interventions with regard to outcomes of public health importance.

## Conclusions

The results of the present study provide quantitative empirical evidence supporting the population-based approach as an effective way to control obesity and may be used as a baseline for further evaluations of the impact of the health policies related to obesity implemented after 2003 in Brazil, based on the Global Strategy on Diet, Physical Activity and Health [14].

## Abbreviations

BMI: Body mass index
HBS: National Household Budget Survey
IBGE: Brazilian Institute of Geography and Statistics
IESC/UFRJ: Institute of Public Health Studies of the Federal University of Rio de Janeiro
